# *APOE* Is Associated With Serum Tau Following Uncomplicated Mild Traumatic Brain Injury

**DOI:** 10.3389/fneur.2022.816625

**Published:** 2022-07-14

**Authors:** Sara M. Lippa, Rael T. Lange, Clifton L. Dalgard, Anthony R. Soltis, Vivian A. Guedes, Tracey A. Brickell, Louis M. French, Jessica Gill

**Affiliations:** ^1^National Intrepid Center of Excellence, Walter Reed National Military Medical Center, Bethesda, MD, United States; ^2^Department of Research, Tramatic Brain Injury Center of Excellence, Silver Spring, MD, United States; ^3^Department of Psychiatry, University of British Columbia, Vancouver, BC, Canada; ^4^Centre of Excellence on Post-traumatic Stress Disorder, Ottawa, ON, Canada; ^5^General Dynamics Information Technology, Falls Church, VA, United States; ^6^Department of Psychiatry, Uniformed Services University of the Health Sciences, Bethesda, MD, United States; ^7^The American Genome Center, Bethesda, MD, United States; ^8^Henry M. Jackson Foundation, Bethesda, MD, United States; ^9^PRIMER, Bethesda, MD, United States; ^10^Division of Intramural Research, National Institute of Nursing Research, National Institutes of Health, Bethesda, MD, United States; ^11^School of Nursing, Johns Hopkins University, Baltimore, MD, United States; ^12^Department of Neurology, School of Medicine, Johns Hopkins University, Baltimore, MD, United States

**Keywords:** traumatic brain injury, APOE, biomarkers, serum, tau, military

## Abstract

**Background and Objectives:**

APOE e4 has been linked to poor outcome following traumatic brain injury (TBI); however, the mechanisms behind this relationship are unclear. Few studies have investigated the relationship between the APOE genotype and established brain related protein biomarkers following TBI. The purpose of this study was to examine this relationship in service members and veterans (SMVs) following TBI.

**Methods:**

Participants were 209 SMVs [124 uncomplicated mild TBI (mTBI); 85 complicated mild, moderate, severe, or penetrating TBI (mod-sev TBI)] prospectively enrolled in the DVBIC-TBICoE 15-Year Longitudinal TBI Study. APOE genotyping was undertaken using non-fasting blood serum samples. Participants were divided into three groups: APOE e2+, APOE e3/e3, and APOE e4+.

**Results:**

In participants with mTBI, those with the APOE e2 allele had significantly lower levels of tau than those with APOE e4 (*p* = 0.005, *r* = 0.43, medium-large effect size). Those with APOE e3/e3 trended toward having higher tau than those APOE e2+ (*p* = 0.076, *r* = 0.20, small-medium effect size) and lower tau than those with APOE e4+ (*p* = 0.062, *r* = 0.21, small-medium effect size). There were no significant differences in biomarkers based on APOE in the mod-sev TBI group.

**Discussion:**

This study is the first to demonstrate APOE genotype is related to serum tau levels following a mTBI, extending prior findings to human serum following mTBI. In addition to higher serum tau levels in APOE e4 carriers, lower tau levels were observed in APOE e2 carriers, suggesting a possible protective effect.

## Introduction

Apolipoprotein E e4 genotype has been linked to poor outcome following traumatic brain injury (TBI) (1); however, the mechanisms behind this relationship are unclear. To our knowledge, few studies have investigated the relationship between APOE genotype and peripherally circulating proteins following TBI. Some proteins of interest include tau, neurofilament light (NfL), glial fibrillary acidic protein (GFAP), and ubiquitin c-terminal hydrolase L1 (UCH-L1), all of which can be measured in blood after TBI. Tau is highly expressed in unmyelinated cortical axons (2). Diffuse axonal injury increases extracellular tau, which can then cross the blood brain barrier and be measured in blood samples. Similar to tau, NfL provides structure to axons, though it is most abundant in myelinated axons projecting subcortically and to the spinal cord (3). Glial Fibrillary Acidic Protein is an intermediate filament III protein highly expressed in astrocytes that maintains structure and strength of glial cells and supports neurons and the BBB. After brain injury, GFAP activates astrogliosis, increasing the size and number of astrocytes (4). Ubiquitin C-terminal hydrolase removes ubiquitin from proteins (5). It is generally found in the neuronal soma cytoplasm. Its presence in the blood therefore indicates injury of the neuronal cell body (6). Decreased UCH-L1 can lead to increased alpha-synuclein in neurodegenerative processes (7, 8).

There is very little research investigating the relationship between APOE genotype and blood-based biomarkers following TBI. In boxers with blood and cerebrospinal fluid (CSF) collected twice within 14 days of a recent bout, APOE genotype was unrelated to blood levels of amyloid-β-42, or CSF levels of phosphorylated neurofilament-heavy chain, amyloid precursor proteins, APOE levels, neurofilament light (NfL), glial fibrillary acidic protein (GFAP), phosphorylated-tau (*p*-tau), or S-100B (9). Thus, there may not be short term protein level changes related to this gene; however, existing evidence suggests APOE genotype may have longer-term impacts on biomarkers.

Supporting this, in Alzheimer's Disease (AD), APOE e4 is the preeminent genetic risk factor (10). Within AD samples, APOE e4 has repeatedly been associated with accelerated neurodegeneration of the medial temporal lobe, reduction in neuronal and synaptic integrity (11). APOE has been linked to amyloid-β (12–18), with APOE e4 detrimental and APOE e2 protective (19). Additionally, a relationship between APOE and tau has become apparent (20). Several studies have demonstrated that APOE e4 modifies tau pathology and neurodegeneration (20–23), including over and above amyloid-β (24). In APOE e4 carriers, CSF levels of APOE have been associated with CSF levels of tau and *p*-tau (25). Additionally, APOE e4-expressing mice had increased tau phosphorylation and learning impairments (26).

Understanding whether APOE impacts brain related protein levels following TBI has implications for current functioning, future neurodegeneration, and treatment targets. This study sought to determine whether blood biomarkers relevant in TBI (tau, NfL, GFAP, and UCH-L1) are elevated in service members and veterans (SMV) with history of TBI with APOE e4 compared to those without APOE e4. It also sought to determine whether SMVs with APOE e2 may have reduced levels of these biomarkers compared to those without APOE e2.

## Materials and Methods

### Participants

Participants were 209 United States SMVs prospectively recruited into the Defense and Veterans Brain Injury Center/Traumatic Brain Injury Center of Excellence 15-Year Longitudinal TBI Study through community events and four Medical Treatment Facilities a year or more following TBI. General exclusion criteria included significant neurological/psychiatric condition(s) unrelated to the injury event.

Participants were selected for inclusion in the final sample if they had a history of TBI, underwent APOE genotyping, and had ≥1 useable biomarker samples. This resulted in 124 uncomplicated mild TBI (mTBI) and 85 complicated mild, moderate, severe, or penetrating TBI (mod-sev TBI).

### TBI Evaluation and Classification

Diagnosis and classification of TBI has been detailed previously (27). A comprehensive lifetime TBI history including the Ohio State University TBI identification method and medical record review was used to classify TBI severity during case conferencing (27). Uncomplicated mTBI (*n* = 124) was defined as: no trauma-related intracranial abnormality (ICA) on CT or structural MRI and either Glasgow Coma Scale (GCS) = 13–15, posttraumatic amnesia (PTA) < 24 h, loss of consciousness (LOC) < 30 mins, or alteration of consciousness present. Complicated mild, moderate, severe, or penetrating TBI (*n* = 85) was defined as: trauma-related ICA on CT or MRI, LOC > 30 mins, PTA > 1 h, or a breach of the cranial vault and/or dura mater by an external object and/or skull fragment.

### Measures and Procedure

#### Laboratory Analyses

Of note, the samples used in this study overlap with those used in our prior work (28–30). Non-fasting blood samples were collected with plastic serum-separating tubes, processed within an hour, and stored at −80°C. Batch assays were conducted after all samples had been collected. Simoa™ (Quanterix, Lexington, MA), a high-definition-1 analyzer, measured biomarker concentrations. Samples were randomized over plates and run in duplicate with laboratory scientists blinded to participant groups, using an ultrasensitive multiplex immunoassay (Neurology 4-Plex A 102153). Blood samples were not used if the reported coefficients of variation (CV) were over 20% and they were above the lower limit of quantification, and therefore two NfL, 46 tau, 85 UCH-L1 samples were excluded. Average CVs were: GFAP-3.6%, NfL-7.1%, tau-14.9%, UCH-L1-33.0%. The lower limits of quantification are: GFAP-0.467 pg/ml, NfL-0.241 pg/ml, tau-0.053pg/ml, UCH-L1-5.45pg/ml.

DNA samples were added to Covaris 96 microTUBE plates at 1,000 ng input and sheared using the Covaris LE220 Focused-ultrasonicator and settings (t: 78; Duty: 18; PIP: 450; 200 cycles) for a peak size of 410 bp. Sequencing libraries were generated using the Illumina TruSeq DNA PCR-Free Library Preparation Kit with robotic automation (Hamilton STAR System) and IDT for Illumina TruSeq DNA UD Indexes (96 Indexes, 96 Samples) adapters. Library size distribution and absence of adapter dimers were confirmed by automated capillary gel-electrophoresis (Advanced Analytical Fragment Analyzer). Library concentration was determined by qPCR using the KAPA qPCR Quantification Kit (Roche Light Cycler 480 Instrument II). Sequencing libraries were pooled at 24-plex and quantified as above before sequencing on an Illumina NovaSeq 6000 using a S4 Reagent Kit (300 cycles) with 151+8+8+151 cycle run parameters. Raw data were demuxed using the Illumina HAS2.2 pipeline and sample-level quality control for base quality, coverage, duplicates, and contamination (FREEMIX <0.05) was conducted.

APOE genotypes were determined through base identities at hg38 positions chr19:44908822 (rs7412; reference C, variant T) and chr19:44908684 (rs429358; reference T, variant C). A two-element tuple of variant allele counts at these positions was generated and converted to APOE genotypes according to: (0,0) = e3/e3, (0,1) = e3/e4, (0,2) = e4/e4, (1,0) = e2/e3, (1,2) = e1/e4, and (2,0) = e2/e2. Genotype (1,1) was considered e2/e4 given the low expected probability of observing the double variant TC allele.

APOE genotyping results for the entire sample was as follows: e4/e4 (2.2%), e3/e4 (24.4%), e3/e3 (55.6%), e2/e4 (1.4%), e2/e3 (16.1%), e2/e2 (0.4%). Participants were divided into one of three groups: APOE e2+ (e2/e2, e2/e3), e3/e3, or APOE e4+ (e3/e4, e4/e4). Those with e2/e4 were excluded from group comparisons.

#### Standard Protocol Approvals, Registrations, and Patient Consents

This research was conducted in accordance with the Declaration of Helsinki guidelines and approved by the Walter Reed National Military Medical Center Institutional Review Board (IRB). Written informed consent was obtained from all participants.

#### Data Availability Statement

Summary/aggregate data and additional information on the methods and statistical analyses will be provided on request. However, individual data elements are not available due to DoD legal requirements and current IRB approved language in the subject consent forms.

## Results

Descriptive statistics for all APOE genotypes are presented in Table 1. Descriptive statistics and group comparisons for demographic, clinical characteristics, and blood biomarkers by APOE group were conducted with ANOVAs, Kruskal Wallis, and Mann-Whitney tests and are presented in Table 2. The APOE e2+ group had a history of sustaining a higher number of lifetime TBIs than the e3/e3 group (*p* = 0.008, *r* = 0.21, small-medium effect size) and the APOE e4+ group (*p* = 0.021, *r* = 0.25, small-medium effect size). Tau was higher in the APOE e4+ group compared to the APOE e2+ group (*p* = 0.008, *r* = 0.31, medium effect size).

**Table 1:** Selected demographics and injury characteristics by APOE genotype.

	**e2/e2 (*****n*** **= 1)**		**e2/e3 (*****n*** **= 33)**		**e2/e4 (*****n*** **= 2)**		**e3/e3 (*****n*** **= 118)**		**e3/e4 (*****n*** **= 50)**		**e4/e4 (*****n*** **= 5)**	
	**Mean**	**SD**	**Mean**	**SD**	**Mean**	**SD**	**Mean**	**SD**	**Mean**	**SD**	**Mean**	**SD**
Age	38.0	na	37.0	9.0	31.0	2.8	37.5	9.7	37.1	9.8	32.4	8.6
Months since injury	37.0	na	88.2	69.1	50.0	18.4	84.0	53.1	90.9	75.6	68.8	36.9
	**Med**	**Range**	**Med**	**Range**	**Med**	**Range**	**Med**	**Range**	**Med**	**Range**	**Med**	**Range**
Number of TBIs	1	na	1	1–4	1	1–1	1	1–3	1	1–3	1	1–2
tau	0.49	na	0.36	0.02–2.42	0.60	0.35–0.85	0.43	0.03–2.54	0.51	0.17–2.03	0.37	0.33–0.45
NfL	6.01	na	7.44	1.78–46.22	9.31	5.21–13.41	6.71	1.08–39.32	7.24	2.69–21.30	6.20	4.79–8.22
GFAP	59.37	na	72.00	25.43–151.26	126.80	75.29–178.31	71.45	28.82–492.10	87.15	36.35–318.96	71.87	52.07–450.61
UCHL1	2.89	na	13.49	0.11–139.57	3.86	3.86–3.86	8.60	0.89–91.34	16.00	2.91–69.49	6.36	2.52–20.15
	***n***	**%**	***n***	**%**	***n***	**%**	***n***	**%**	***n***	**%**	***n***	**%**
Male	1	100	31	93.9	2	100	112	94.9	45	90.0	5	100
mTBI	1	100	23	69.7	0	0.0	73	61.9	24	48.0	3	60.0
White	1	100	7	21.2	1	50.0	24	20.3	14	28.0	0	0.0

*APOE, apolipoprotein E; GFAP, glial fibrillary acidic protein; mTBI, uncomplicated mild TBI; NfL, neurofilament light; Mod-Sev TBI, complicated mild, moderate, severe, and penetrating TBI; TBI, traumatic brain injury; UCH-L1, ubiquitin carboxyl-terminal hydrolase L1*.

**Table 2:** Demographics and injury characteristics by APOE allele group.

	**APOE e2+ (*****n*** **= 34)**		**APOE e3/e3 (*****n*** **= 118)**		**APOE e4+ (*****n*** **= 55)**		**F**	***p***
	**M**	**SD**	**M**	**SD**	**M**	**SD**		
Age	37.00	8.88	37.51	9.68	36.65	9.70	0.158	0.854
Years of Education	15.15	2.27	14.97	2.26	14.76	1.91	0.344	0.710
Time Since Injury (months)	86.68	68.65	84.00	53.15	88.87	73.01	0.122	0.885
	**Med**	**IQR**	**Med**	**IQR**	**Med**	**IQR**	**Kruskal Wallis H**	***p***
Number of TBIs	1	1–2	1	1–1	1	1–1	7.892	0.019
Tau	0.369	0.185–0.487	0.426	0.298–0.573	0.494	0.351–0.709	8.394	0.015
NfL	6.99	4.92–10.00	6.71	4.88–9.71	7.20	5.51–9.22	0.325	0.850
GFAP	71.59	59.32–121.45	71.45	59.58–97.12	84.90	58.55–121.16	2.670	0.263
UCH-L1	13.18	4.76–22.36	8.60	4.65–16.66	15.31	4.63–28.52	4.127	0.127
	***n***	**%**	***n***	**%**	***n***	**%**	**Fisher's Exact Test**	***p***
Men	32	94.1	112	94.9	50	90.9	1.18	0.527
White	26	76.5	94	79.7	41	74.5	0.71	0.717
Branch		0.0		0.0		0.0	9.754	0.247
Air Force	1	2.9	9	7.6	2	3.6		
Army	21	61.8	86	72.9	36	65.5		
Navy	6	17.6	10	8.5	10	18.2		
Marines	5	14.7	13	11.0	7	12.7		
Other	1	2.9	0	0.0	0	0.0		
Enlisted	23	67.6	83	70.3	43	78.2	1.549	0.465
TBI severity						4.397	0.115
mTBI	24	70.6	73	61.9	27	49.1		
Mod-Sev TBI	10	29.4	45	38.1	28	50.9		

*APOE, apolipoprotein E; GFAP, glial fibrillary acidic protein; mTBI, uncomplicated mild TBI; NfL, neurofilament light; Mod-Sev TBI, complicated mild, moderate, severe, and penetrating TBI; TBI, traumatic brain injury; UCH-L1, ubiquitin carboxyl-terminal hydrolase L1*.

Kruskal-Wallis H and Mann-Whitney U tests compared biomarkers between the APOE e2+, e3/e3, and APOE e4+ groups within each injury group. Within mTBI participants, the APOE e2+ group had significantly lower levels of tau than the APOE e4+ group (*p* = 0.005, *r* = 0.43, medium-large effect size). Additionally, the e3/e3 group trended toward having higher tau than the APOE e2+ group (*p* = 0.076, *r* = 0.20, small-medium effect size) and lower tau than the APOE e4+ group (*p* = 0.062, *r* = 0.21, small-medium effect size). There were no significant differences in the omnibus tests for biomarkers in the mod-sev TBI group (Table 3).

**Table 3:** Levels of blood biomarkers within each injury group by APOE allele group.

	**1. APOE e2+**		**2. APOE e3/e3**		**3. APOE e4+**		**r1-2**	**r1-3**	**r2-3**	***p*** **overall**	**p1-2**	**p1-3**	**p2-3**
	**Med**	**IQR**	**Med**	**IQR**	**Med**	**IQR**							
**mTBI**													
Tau	0.352	0.161–0.486	0.42	0.299–0.573	0.524	0.348–0.716	0.20	0.43	0.21	0.013	0.076	0.005	0.062
NfL	6.99	4.75–10.55	6.69	4.88–9.38	7.48	5.70–9.33	0.05	0.05	0.10	0.607	0.658	0.733	0.322
GFAP	69.97	56.57–106.86	69.55	56.61–81.90	70.73	56.23–100.05	0.05	0.03	0.00	0.910	0.646	0.806	0.972
UCH-L1	13.18	4.76–21.95	10.44	3.77–19.49	14.18	3.59–32.94	0.09	0.07	0.13	0.546	0.502	0.692	0.315
**Mod-Sev TBI**													
Tau	0.399	0.162–1.19	0.444	0.284–0.583	0.436	0.351–0.726	0.06	0.11	0.08	0.753	0.689	0.576	0.549
NfL	7.32	5.04–9.98	7.06	4.85–10.66	6.5	5.14–8.85	0.01	0.05	0.04	0.912	0.929	0.740	0.703
GFAP	116.76	65.88–123.82	86.33	60.47–118.30	109.62	80.09–142.74	0.12	0.06	0.18	0.249	0.371	0.715	0.115
UCH-L1	14.50	4.96–110.04	8.16	4.97–12.06	16.69	5.66–27.74	0.14	0.02	0.33	0.158	0.486	0.920	0.049

*APOE, apolipoprotein E; GFAP, glial fibrillary acidic protein; mTBI, uncomplicated mild TBI; NfL, neurofilament light; Mod-Sev TBI, complicated mild, moderate, severe, and penetrating TBI; TBI, traumatic brain injury; UCH-L1, ubiquitin carboxyl-terminal hydrolase L1*.

## Discussion

This study is the first to demonstrate APOE alleles relate to serum tau levels following mTBI in humans, suggesting APOE e4 may increase serum tau while APOE e2 may decrease it. These findings are consistent with prior basic research (20–24). This differential impact of APOE on tau in patients with a history of mTBI may have implications for future neurodegeneration, and warrants additional investigation. Pre-clinical research also supports this hypothesis, with a mouse model demonstrating increased tau phosphorylation and learning impairments in APOE e4-positive mice (26). It is possible that APOE e4 is associated with increased tau distribution to the medial temporal lobes (24), negatively impacting memory. We previously demonstrated, in overlapping but non-identical cohorts, that APOE e4 is related to worse memory performance in SMVs a year or more after TBI (30). In separate studies we have not found a cross-sectional relationship between serum tau and cognition within the first year following a TBI (29) or more than a year after TBI, (28) though we did find that serum tau within the first year of a TBI predicted decline in perceptual reasoning and executive functioning over time (29). Future investigation into how tau may mediate the impact of APOE on cognition and clinical status following TBI is warranted.

Interestingly, individuals with an APOE e2 allele sustained more TBIs than individuals without an APOE e2 allele. This is in contrast to past research finding no difference in e2 alleles between college athletes with multiple concussions and those with zero or one concussions (31). It is possible that the APOE e2 allele increases one's risk of sustaining a higher number of lifetime TBIs than the e3/e3 group; however, it is notable that the vast majority of prior studies have not found a relationship between APOE genotype and risk of TBI (32–34). Some of these studies have focused on the APOE e4 allele (35–37), while others have included an investigation of APOE e2 (31, 38, 39).

Notably, there was no relationship between APOE and tau (or other biomarkers) in the mod-sev TBI group, suggesting that mTBI and more severe TBI may result in different biological processes a year or more post-injury. The lack of relationship could also be due to small sample size, especially in the APOE e2+ group, or limited examination of four serum biomarkers. The present findings and discrepancy between the mild and mod-sev TBI group suggest additional research is required to understand how APOE genotype impacts biomarker levels following TBI of different severity.

Additional limitations include the lack of consideration of serum amyloid-β levels. Traditionally, APOE e4 has been associated with increased levels of amyloid-β (2–18). We previously did not find a relationship between amyloid-β levels and TBI severity, diffusion tensor imaging, or cognitive performance in a subsample of these participants (27). Though serum amyloid-β is not currently available on the entire sample, it seems likely that a similar, and perhaps stronger, relationship between APOE and amyloid-β would be observed compared to the relationship we demonstrated between tau and APOE.

Despite these limitations, this study builds on prior findings showing APOE genotype is related to tau pathology (20, 24, 26). We found that APOE genotype was related to serum tau in patients with mild TBI, with APOE e2 associated with lower serum tau levels and APOE e4 associated with higher serum tau levels; however, this relationship between APOE and tau did not exist in the moderate-severe TBI group. Findings require replication in larger samples with clinical relevance explored longitudinally.

## Data Availability Statement

The data analyzed in this study is subject to the following licenses/restrictions: summary/aggregate data and additional information on the methods and statistical analyses will be provided on request. However, individual data elements are not available due to Department of Defense (DoD) legal requirements and current Institutional Review Board (IRB) approved language in the subject consent forms. Requests to access these datasets should be directed to RL, rael.lange@gmail.com.

## Ethics Statement

The studies involving human participants were reviewed and approved by Walter Reed National Military Medical Center Institutional Review Board. The patients/participants provided their written informed consent to participate in this study.

## Author Contributions

SL designed and conceptualized study, data collection, data analysis, and drafted the manuscript for intellectual content. RL, LF, and TB designed and conceptualized the study, data collection, and revised the manuscript for intellectual content. CD, AS, VG, and JG data analysis and revised the manuscript for intellectual content. All authors contributed to the article and approved the submitted version.

## Funding

This study was supported by the Traumatic Brain Injury Center of Excellence and was designed to respond to a Congressional mandate (Sec721 NDAA FY2007). This work was prepared under Contract HT0014-19-C-0004 with DHA Contracting Office (CO-NCR) HT0014 and, therefore, is defined as U.S. Government work under Title 17 U.S.C.§101. Per Title 17 U.S.C.§105, copyright protection is not available for any work of the U.S. Government. For more information, please contact dha.TBICoEinfo@mail.mil.

## Author Disclaimer

The identification of specific products or scientific instrumentation is considered an integral part of the scientific endeavor and does not constitute endorsement or implied endorsement on the part of the author(s), DoD, or any component agency. The views expressed in this manuscript are those of the author(s) and do not necessarily reflect the official policy of the Department of Defense or the U.S. Government.

## Conflict of Interest

AS was employed by Henry M. Jackson Foundation and PRIMER. The remaining authors declare that the research was conducted in the absence of any commercial or financial relationships that could be construed as a potential conflict of interest.

## Publisher's Note

All claims expressed in this article are solely those of the authors and do not necessarily represent those of their affiliated organizations, or those of the publisher, the editors and the reviewers. Any product that may be evaluated in this article, or claim that may be made by its manufacturer, is not guaranteed or endorsed by the publisher.
